# Unique features of the rice blast resistance *Pish *locus revealed by large scale retrotransposon-tagging

**DOI:** 10.1186/1471-2229-10-175

**Published:** 2010-08-13

**Authors:** Akira Takahashi, Nagao Hayashi, Akio Miyao, Hirohiko Hirochika

**Affiliations:** 1 Plant Disease Resistance Research Unit, National Institute of Agrobiological Sciences, Ibaraki 305-8602, Japan; 2 Division of Genome and Biodiversity Research, National Institute of Agrobiological Sciences, Ibaraki, 305-8602, Japan

## Abstract

**Background:**

*R *gene-mediated resistance is one of the most effective mechanisms of immunity against pathogens in plants. To date some components that regulate the primary steps of plant immunity have been isolated, however, the molecular dissection of defense signaling downstream of the R proteins remains to be completed. In addition, *R *genes are known to be highly variable, however, the molecular mechanisms responsible for this variability remain obscure.

**Results:**

To identify novel factors required for *R *gene-mediated resistance in rice, we used rice insertional mutant lines, induced by the endogenous retrotransposon *Tos17*, in a genetic screening involving the rice blast fungus *Magnaporthe oryzae*. We inoculated 41,119 mutant lines with the fungus using a high throughput procedure, and identified 86 mutant lines with diminished resistance. A genome analysis revealed that 72 of the 86 lines contained mutations in a gene encoding a nucleotide binding site (NBS) and leucine rich repeat (LRR) domain-containing (NBS-LRR) protein. A genetic complementation analysis and a pathogenesis assay demonstrated that this NBS-LRR gene encodes Pish, which confers resistance against races of *M. oryzae *containing *avrPish*. The other 14 lines have intact copies of the *Pish *gene, suggesting that they may contain mutations in the signaling components downstream of Pish. The genome analysis indicated that *Pish *and its neighboring three NBS-LRR genes are high similar to one another and are tandemly located. An *in silico *analysis of a *Tos17 *flanking sequence database revealed that this region is a "hot spot" for insertion. Intriguingly, the insertion sites are not distributed evenly among these four NBS-LRR genes, despite their similarity at the sequence and expression levels.

**Conclusions:**

In this work we isolated the *R *gene *Pish*, and identified several other mutants involved in the signal transduction required for *Pish*-mediated resistance. These results indicate that our genetic approach is efficient and useful for unveiling novel aspects of defense signaling in rice. Furthermore, our data provide experimental evidence that *R *gene clusters have the potential to be highly preferred targets for transposable element insertions in plant genomes. Based on this finding, a possible mechanism underlying the high variability of *R *genes is discussed.

## Background

Plants have evolved elaborate defense mechanisms to protect themselves from many kinds of pathogens, including fungi, bacteria, viruses, and insects. Defense responses governed by the gene-for-gene hypothesis are triggered in plants when the product of a plant resistance (*R*) gene directly or indirectly recognizes a specific pathogen effector molecule, which is often the product of a pathogen avirulence (*avr*) gene [1]. The absence or inactivation of either member of this gene pair results in susceptibility of the host to the pathogen. To date more than 40 *R *genes have been isolated from several plant species, and most of them exhibit highly conserved structures, despite differences between the types of pathogens that are recognized. Pathogen recognition by any R protein initiates a common set of defense responses, including the production of reactive oxygen species (ROS), expression of pathogen-related (*PR*) genes, and localized programmed cell death at the site of pathogen challenge, which is known as the hypersensitive response (HR) [2]. This suggests that common downstream components may be shared by R proteins within and among plant species.

The most prevalent class of plant R proteins contains a nucleotide binding site (NBS) and a leucine-rich repeat (LRR) region, and are thus called NBS-and-LRR containing (NBS-LRR) proteins. The NBS domain is a functional ATPase and probably regulates the activity of the R proteins [3]. The LRR domain is required for specific recognition of the pathogen containing corresponding avirulence gene, and changing even a single amino acid in this region alters its recognition specificity, resulting in disease [4]. In addition, there are reports that the N-terminal region of the Toll protein and interleukin-1 receptor (TIR) domain and the putative coiled-coil (CC) domain of some NBS-LRR proteins are involved in specific pathogen recognition [5,6]. Genome analyses have revealed that a large number of NBS-LRR genes exist in plant genomes. The Arabidopsis and rice genomes contain up to 150 and 600 NBS-LRR genes, respectively, and many of them are tightly clustered [1,7].

Rice blast, caused by *Magnaporthe oryzae*, is one of the most serious diseases of rice world-wide. Many *R *genes against the fungus have been genetically mapped and some of them have been utilized for breeding in rice [8]. To date, 12 blast *R *genes have been isolated by map-based cloning, and 11 of these encode NBS-LRR resistance proteins [4,9-18]. The Japonica rice cultivar Nipponbare (NB) has been selected as a standard cultivar for the international rice genome sequencing project. Therefore, NB is a strong tool for the genetic analysis of defense signaling in rice. NB carries *R *gene-mediated resistance against *M. oryzae *containing *avrPish *[19]. Although the *Pish *locus has been mapped on chromosome 1 by an analysis of quantitative trait loci (QTL), the gene itself has been difficult to isolate because of it confers only moderate resistance [20,21]. The isolation of *R *genes from NB is important for the molecular dissection of defense mechanisms mediated by R proteins in rice.

The functional and structural diversification of *R *genes is crucial for the survival of plants, which must fight off rapidly evolving pathogens. The molecular evolution of *R *genes is thought to be affected by several mechanisms, including mutations caused by transposable elements (TEs). Many types of TEs have been identified in *R *gene clusters [22,23]. TE-mediated genome reorganization and TE-associated methylation may play roles in *R*-gene evolution, in addition to the actual insertion of TEs. Therefore, target-site selection by TEs is of great interest in the study of *R *gene evolution. Many TEs target specific chromosomal sites [24]. For some elements, the target sites are determined by specific DNA sequences, whereas for others, including several retrotransposons and retroviruses, chromatin structure impacts the choice of target site. The large-scale analysis of sequences flanking the rice retrotransposon *Tos17 *indicated that it prefers gene-dense regions over centromeric heterochromatin regions [25].

Here we report on our screening approach to identifying signaling components required for *R *gene-mediated disease resistance, using a collection of NB-background mutant lines generated by activation of the rice endogenous retrotransposon *Tos17 *[26]. We identified many mutant lines with reduced resistance to *M. oryzae *containing *avrPish*. While most of the mutants contained *Tos17 *insertions or deletions in the *Pish *gene locus, several contained intact copies of the *Pish *gene. A genome analysis revealed that *Pish *belongs to the NBS-LRR class of *R *genes, and three highly conserved NBS-LRR genes lie near it. Interestingly, this gene cluster is one of the hot spots of *Tos17 *insertion, and the target sites are concentrated within the *Pish *locus despite its similarity to the other genes at the sequence and expression levels. This suggests that the preference for *Tos17*-insertion in the *Pish *locus is regulated at the chromatin level.

## Results

### Large scale genetic screening

**Figure 1:**
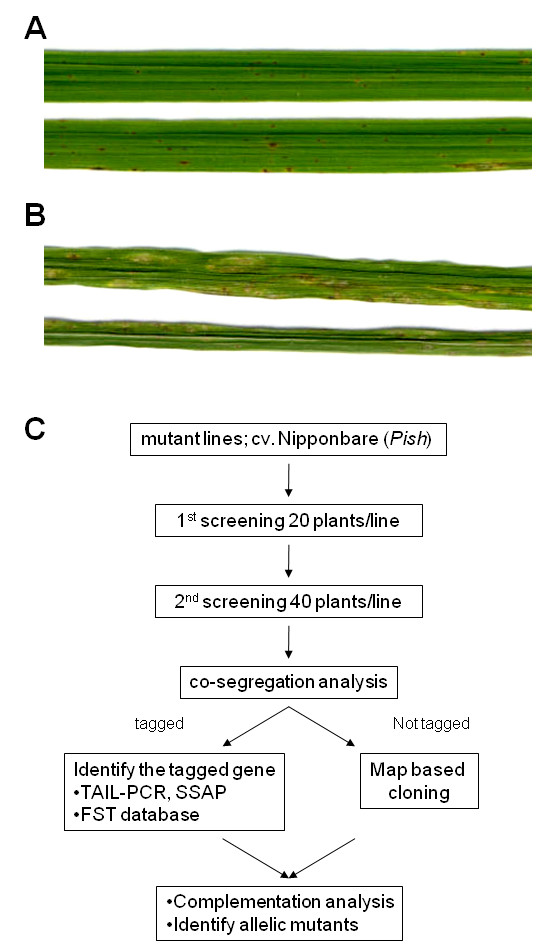
**High throughput screening of rice mutant lines**. Representative phenotypes of NB (A) and KM (B) leaves inoculated with the rice blast fungus *Magnaporthe oryzae*, isolate Kyu77-07A (*avrPish*; Race 102.0). Photographs were taken 7 days post inoculation. (C) Experimental scheme for high throughput screening of *Tos17 *insertional mutant lines.

To efficiently screen for loss-of-function mutations in genes encoding components of disease resistance signal transduction, we used insertion mutant lines of NB in which the endogenous retrotransposon *Tos17 *had been activated by tissue culture [26,27]. While NB carries the *Pish *gene [19], cv. Kinmaze (KM) does not and is susceptible to the same race of the fungus (Figure 1A, B). To perform high throughput screening, we inoculated 3 week-old seedlings with the fungal isolate containing *avrPish*. In the first screen, 20 R1 segregating plants per line from 41,119 mutant lines were inoculated. Mutant candidates with susceptible phenotypes (561 lines) were selected for further screening. In the second screen, about 40 plants per selected line were inoculated with the same race of the fungus. We finally identified 86 mutant lines with susceptible phenotypes. These mutant lines were subjected to DNA gel blot analysis to examine co-segregation of *Tos17 *with the mutant phenotype. Genomic DNA sequences flanking the co-segregating *Tos17 *insertions were isolated by thermal asymmetric interlaced PCR (TAIL-PCR) [28], or by identifying sequence-specific amplification polymorphisms [29]. Additional flanking sequences were identified by searching the flanking sequence tag (FST) database [30]. In cases where no co-segregating signal was obtained, the causative genes will be isolated by a map-based cloning strategy (Figure 1C).

### Isolation of *pish *mutants

**Figure 2:**
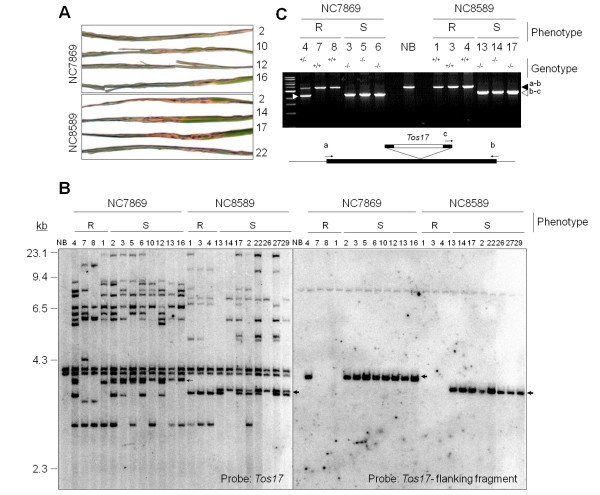
**Co-segregation analysis of mutant lines NC7869 and NC8589**. (A) Representative phenotypes of the susceptible mutants that segregated in these lines. Numbers on the right of the photographs represent individual seedlings. (B) DNA gel blot analysis of *Xba*I-digested genomic DNA was carried out to investigate co-segregation between disease phenotypes and *Tos17 *insertions in individual plants of the NC7869 and NC8589 lines. Disease phenotypes are indicated at the top as R (resistant) and S (susceptible) to the blast fungus containing *avrPish*. Numbers at the top of each lane represent individual seedlings. NB served as a control for wild-type (resistant) seedlings. The blot was probed first with a ^32^P labeled *Tos17 *fragment (left) and then with a labeled fragment flanking the co-segregating *Tos17 *copy (right). The positions of size markers (λ DNA digested with *Hin*dIII) are shown on the left. Co-segregating bands are indicated by arrowheads. (C) Genotyping PCR analysis of genomic DNA from NC7869 and NC8589 plants exhibiting resistance (R) or susceptibility (S). The black triangle indicates the approximately 3.6 kb fragment that was amplified from the wild-type genome using primers at positions indicated by a and b on the diagram below the PCR gel. The white triangles indicate the fragments amplified using primers at positions b and c, designed to amplify the flanking sequence of *Tos17*. The genotype of each seedling is given at the top of each lane.

**Figure 3:**
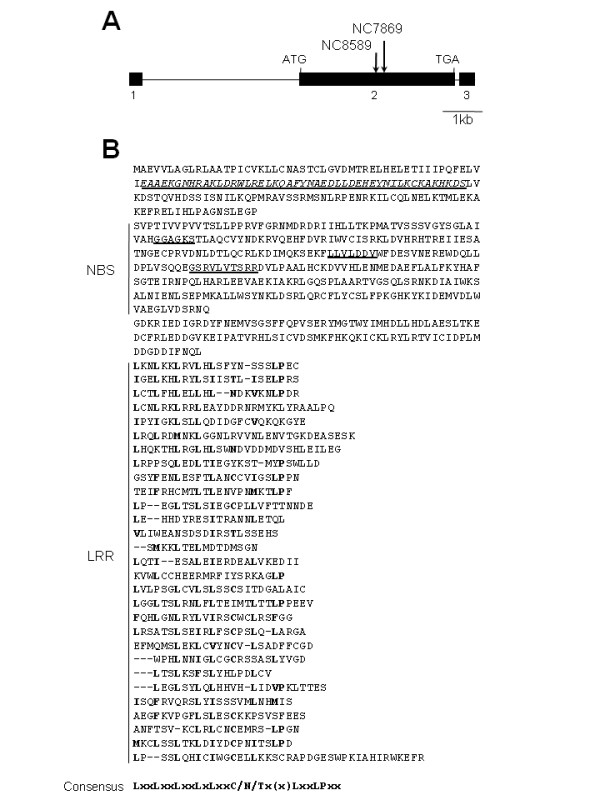
**The structure of the *Pish*(t) gene and its deduced amino acid sequence**. (A) Positions of *Tos17 *insertions within the *Pish*(t) gene in mutant lines NC7869 and NC8589. Exons are indicated by black boxes. (B) Deduced amino acid sequence of the *Pish*(t) gene product. Amino acids 52-98 shown in underlined italics, constitute a predicted CC motif. The three conserved motifs in the NBS region are also underlined.

We analyzed in detail two mutant lines, NC7869 and NC8589, and observed the segregation of individuals exhibiting complete susceptibility to the rice blast isolate containing *avrPish *in the R1 population of these lines (Figure 2A). The uninfected mutants showed no obvious morphological phenotypes compared with their wild-type siblings (data not shown). Segregants of these lines were screened for the presence of *Tos17 *insertions. Sixteen and 11 newly transposed copies of *Tos17 *were detected in the NC7869 and NC8589 lines, respectively, in addition to the two original copies (Figure 2B, left panel). One of the transposed copies (indicated by arrowheads in Figure 2B) was shown to co-segregate with the susceptible mutant phenotype in each line. Genomic sequences flanking the co-segregating *Tos17 *copies were amplified by TAIL-PCR and used as probes on the same membrane. One of the probes hybridized with both the 4.2-kb band in NC7869 and the 3.9-kb band in NC8589 (Figure 2B, right panel). Because the size of the *Xba*I fragment detected by this probe was 1.3 kb if *Tos17 *was not inserted, the wild-type band was not detected in this analysis. Therefore, we carried out a PCR analysis to examine whether the inheritance pattern of the *Tos17 *insertion was correlated with the susceptibility phenotype. In all of the susceptible plants, homozygous insertions were detected, as indicated by the amplification of only a smaller fragment assumed to be the product of the *Tos17 *specific primer and one of the flanking sequence primers (Figure 2C). On the other hand, all of the wild-type siblings were heterozygous or did not contain the insertion. These results strongly suggested that the susceptibility phenotype of the NC7869 and NC8589 lines was caused by the *Tos17 *insertion, and that the insertional mutation was inherited recessively. A BLAST search of the GenBank nucleotide database and the Rice Annotation Project (RAP) database [31] with the flanking sequence revealed that *Tos17 *was inserted into the 2nd exon of Os01g0782100 in both lines (Figure 3A). This gene contains two introns of 3947 and 109 bp. It encodes a predicted polypeptide of 1290 amino acids with a molecular weight of 147 kDa, which contains conserved NBS and LRR domain motifs (Figure 3B). The NBS domain (169-479 a.a.) contains three sequences, GGAGKS, LLVLDDV, and GSRVLVTSRR, which correspond to the conserved kinase 1a (P-loop), kinase 2, and kinase 3a domains, respectively [32]. The C-terminal region of the protein is composed of 29 irregular LRRs. Further analysis using the Paircoil2 program [33,34] revealed a potential CC domain with a threshold of 0.1 near the N-terminus, indicating the possibility that the encoded protein belongs to the CC-NBS-LRR subclass of R proteins (Figure 3B). Os01g0782100 is located on the long arm of chromosome 1, where *Pish *has been mapped between the markers RM212 (7.2 cM) and OSR3 (15.2 cM) by QTL analysis [20,21]. Taken together, the results suggest that Os01g0782100 is *Pish*, and we designated it *Pish*(t).

### Isolation of allelic mutants and complementation analysis

**Figure 4:**
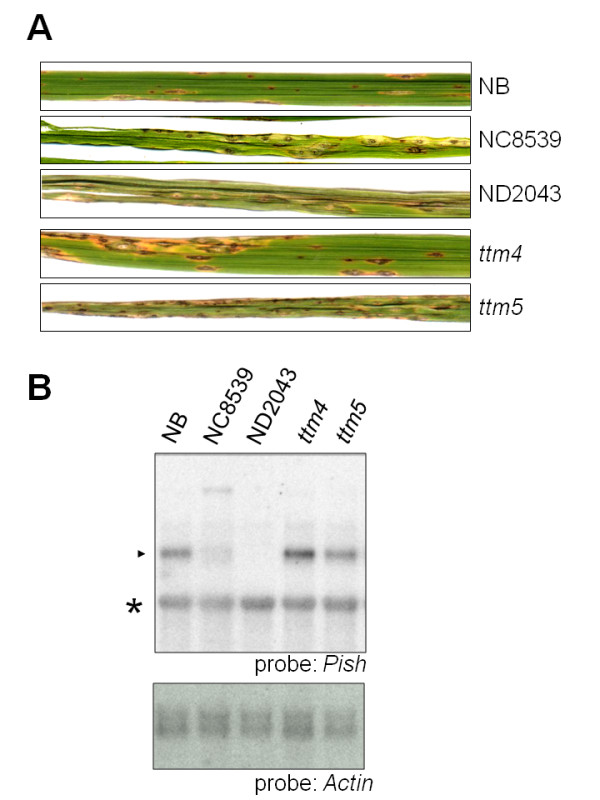
**The susceptible phenotype of selected mutant lines**. (A) Three-week-old NB, *pish *mutant lines, NC8589 andND2043, and *ttm *mutant lines were inoculated with *M. oryzae *containing *avrPish*. The photographs were taken 7 days post inoculation. (B) Expression of the *Pish *gene. Total RNA was extracted from leaf tissues of NB and each mutant. Samples (1 μg) of poly(A)^+ ^RNA were separated by gel electrophoresis, blotted, and hybridized with radiolabeled probes as indicated. The *Actin *probe was used as a loading control. The closed arrowhead indicates the size of the *Pish *mRNA. The asterisk indicates a non-specific signal detected by the *Pish *probe.

To confirm whether the null mutation of *Pish*(t) caused disruption of the resistance mediated by Pish, we screened for other allelic mutants among the 86 selected mutants from our screening. As expected, most of the mutants (72 of the 86 lines) had mutations caused by *Tos17 *insertions, deletions, or an unknown insertion in this locus. Because these mutant lines were produced by tissue culture and are derived from a relatively small number of induced calli, some of these mutations are shared in multiple independently regenerated plants [27]. By considering which calli the mutants were derived from and by examining each mutation pattern (i.e., the positions of *Tos17 *insertions or the deletion sizes in those mutants), we determined that there are 56 independent mutant alleles at the *Pish*(t) locus among the 72 mutant lines (Table 1). Of these, 46 alleles were caused by *Tos17 *insertion. The direction of *Tos17 *was not always the same and the insertion sites were dispersed evenly at this locus, suggesting that the insertion site within the locus was random rather than depending on specific DNA sequences. Nine independent deletion mutations were detected among thirteen lines. The deletion sizes were diverse, ranging from 24 bp to over 50 kb. One allele in the ND2032/ND2105/ND2452/ND2562 lines contained an unidentified insert of about 2.5-kb. In these mutants, the transcription of *Pish*(t) was barely detectable or not detected at all (typical examples are shown in Figure 4A, B).

**Table 1:** Allele of *pish*(t) obtained in this screening

	Line Name	Mutation	Direction^a)^	Position^b)^	Origin^c)^
1	NC7869	*Tos17 *insertion	↑	+2.4	N0
2	NC8589	*Tos17 *insertion	↑	+2.2	N0
3	ND0085	*Tos17 *insertion	↓	+1.1	N1
4	ND2294	*Tos17 *insertion	↓	+2.8	N3
5	ND2607	*Tos17 *insertion	↓	+1.7	N3
6	ND2794	*Tos17 *insertion	↓	+3.6	N3
7	ND3377	*Tos17 *insertion	↑	+2.4	N4
8	ND3760	*Tos17 *insertion	↑	+1.5	N4
9	ND5163	*Tos17 *insertion	↑	+1.1	N7
10	ND5216/ND5490/ND5546	*Tos17 *insertion	↑	+1.3	N7
11	ND5907	*Tos17 *insertion	↑	+1.0	N7
12	ND6103	*Tos17 *insertion	↓	+2.7	N8
13	ND6886	*Tos17 *insertion	↑	+1.0	N8
14	ND7244/ND7372	*Tos17 *insertion	↑	+0.4	N9
15	ND7350/ND7813	*Tos17 *insertion	↓	-0.4	N9
16	ND7933	*Tos17 *insertion	↓	+3.7	N9
17	ND9375	*Tos17 *insertion	↓	+2.9	N12
18	ND9468	*Tos17 *insertion	↓	+1.3	N12
19	NE0979	*Tos17 *insertion	↓	+0.7	N13
20	NE1033	*Tos17 *insertion	↑	+3.5	N14
21	NE1136	*Tos17 *insertion	↓	+0.1	N14
22	NE1348	*Tos17 *insertion	↓	+2.4	N14
23	NE1445	*Tos17 *insertion	↑	+3.3	N14
24	NE1544/NE1588/NE1602/NE1617	*Tos17 *insertion	↓	+2.1	N15
25	NE1662	*Tos17 *insertion	↓	+1.0	N15
26	NE1710/NE1864	*Tos17 *insertion	↓	+2.4	N15
27	NE2127	*Tos17 *insertion	↓	+3.4	N16
28	NE3788	*Tos17 *insertion	↑	-0.9	N18
29	NE3814	*Tos17 *insertion	↑	+2.3	N18
30	NE9963	*Tos17 *insertion	↓	+3.4	N19
31	NF1237	*Tos17 *insertion	↑	+3.4	N28
32	NF1437/NF1457	*Tos17 *insertion	↓	+0.6	N28
33	NF2942	*Tos17 *insertion	↑	+3.6	N39
34	NF6474	*Tos17 *insertion	↑	+2.4	N33
35	NF6868	*Tos17 *insertion	↓	+2.0	N44
36	NF7813	*Tos17 *insertion	↑	+1.4	N42
37	NF7959	*Tos17 *insertion	↓	+2.2	N42
38	NG0430	*Tos17 *insertion	↑	+1.9	N47-2
39	NG2085	*Tos17 *insertion	↑	+1.7	N51
40	NG3372	*Tos17 *insertion	↑	+2.8	N54
41	NG3664	*Tos17 *insertion	↓	+2.8	N55
42	NG5334	*Tos17 *insertion	↑	-2.4	N61
43	NG6314	*Tos17 *insertion	↓	+3.9	N65
44	NG7885	*Tos17 *insertion	↑	+2.6	N68
45	NG8190	*Tos17 *insertion	↓	+3.3	N69
46	NG8193	*Tos17 *insertion	↓	+2.3	N69
47	NC7524/NC8928	Large deletion			N0
48	NC7925	Large deletion			N0
49	ND2043	Large deletion			N3
50	ND2602/ND2810	330bp deletion			N3
51	ND6227	Large deletion			N8
52	ND8614	Large deletion			N11
53	NE2386	24bp deletion			N16
54	NF4922/NF4946/NF4950	56bp deletion			N41
55	NG2263	Large deletion			N52
56	ND2032/ND2105/ND2452/ND2562	Unknown insertion			N3

a) Downward arrows indicate insertions with *Tos17 *oriented in the same direction as *Pish *and the *Npi37-1 *to *Npi37-3 *genes. Upward arrows indicate insertions in the opposite orientation.

b) Positions (kb) of *Tos17 *insertions relative to the *Pish *ATG initiation codon.

c) Name of the flasks in which their origin calli were cultured.

On the other hand, 14 of the 86 lines with diminished *Pish*-mediated resistance contained neither mutations, insertions, nor deletion in this locus (data not shown; typical examples are shown in Figure 4A). In these mutants, the expression of *Pish*(t) was no different from in the wild type plants (typical examples are shown in Figure 4B). Therefore, we concluded that they are not *pish*(t) mutants and designated them *ttm *(tissue-culture triggered mutation). Although we cannot exclude the possibility that some of the *ttm *mutants have mutations in unknown *R *genes that correspond to the blast isolate carrying additional *avr *genes other than *avrPish*, others are likely to have mutations in components required for activation of *Pish*-mediated disease resistance.

**Figure 5:**
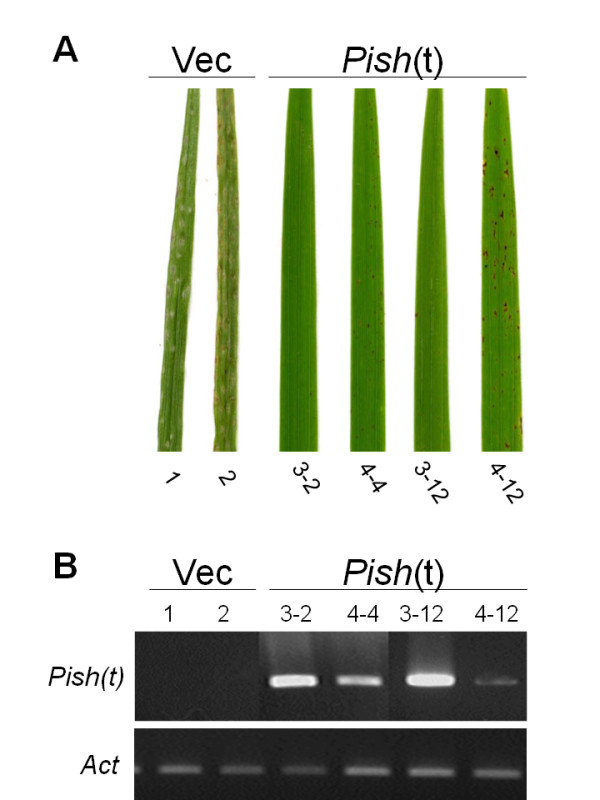
**Complementation tests of transgenic plants**. (A) A cDNAs of *Pish*(t) under the control of the 35 S promoter was introduced into KM. Control plants were transformed with the empty vector (Vec). Three-week-old plants of the T1 and T2 generations were challenged with a rice blast isolate containing *avrPish *by spray inoculation. The photographs show typical phenotypes of leaf blades 7 days post inoculation. Results were reproduced in at least three independent infection experiments using five or more plants each of three independent transgenic lines. (B) RT-PCR analysis was performed on total RNA isolated from leaves of T1 plants transformed with the empty vector or *Pish*(t). An *Action *gene was used as a control for RNA template amounts.

To examine whether Pish(t) confer race-specific resistance, we transformed KM plants with an empty vector control and a construct containing the *Pish*(t) cDNA under the control of the cauliflower mosaic virus 35 S promoter (Figure 5). We obtained more than five independent transgenic lines for each construct, and used the T1 and T2 generations for the following analyses. Transgenic plants containing the *Pish*(t) construct were as healthy as KM plants transformed with the empty vector (data not shown). When infected with a rice blast isolate containing *avrPish*, three independent transgenic lines expressing *Pish*(t) exhibited a resistance phenotype, whereas the lines containing the empty vector were susceptible (Figure 5A and 5B). Thus, expression of the *Pish*(t) cDNA conferred *Pish*-mediated resistance on KM. To determine the resistance spectrum of Pish(t), the transgenic lines were inoculated with seven additional rice blast isolates (Table 2). As expected, the transgenic KM plants containing *Pish*(t) exhibited the same pattern of resistance specificity as the donor cultivar NB. Thus, we concluded that *Pish*(t) is the *Pish *gene.

**Table 2:** Disease reactions of transgenic KM and NB plants to eight *M. oryzae *isolates

		Transgenic lines	
		
Isolate	NB (*Pish*)	**Vector**^**a)**^	***Pish***^**a)**^
Kyu77-07A	R^b^	S^b^	R
84-81A	R	S	R
H07-1-1	S	S	S
Kyu89-246	S	S	S
Ina86-137	S	S	S
TH68-126	S	S	S
24-22-1-1	S	S	S
Kyu9439013	S	S	S

a) The constructs were transformed into the susceptible cultivar KM. Three independent transgenic lines for each construct were assayed.

b) R: resistance, S: susceptible

### Expression analysis of *Pish*

**Figure 6:**
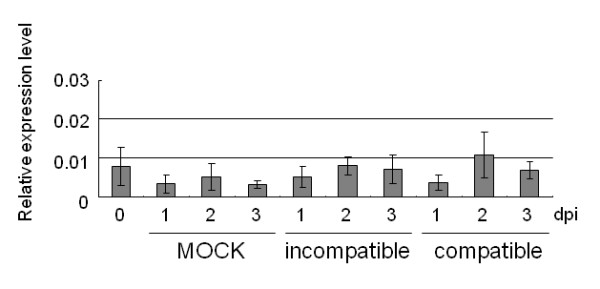
**Quantitative real-time RT-PCR analysis of *Pish *expression**. Total RNA was isolated from NB leaves at 0, 1, 2, and 3 days after inoculation with incompatible or compatible races of *M. oryzae*, or after mock inoculations. The samples were quantified using *Actin *mRNA as a reference. Error bars indicate standard deviations.

Quantitative real-time RT-PCR analysis was carried out to investigate the expression pattern of *Pish *after infection with various *M. oryzae *races. The analysis revealed that there were no distinguishable alterations in *Pish *expression levels at different time points after inoculation with either incompatible or compatible races of the fungus, or after mock treatment (Figure 6). This result is consistent with previous reports that other *Pi *genes were constitutively expressed and not induced by pathogen challenge [10,11,13-15]. It is likely that most *Pi *genes are expressed before pathogen invasion and are post-transcriptionally regulated for activation of the signal transduction pathways leading to resistance responses.

### The genomic organization in the *Pish *region

**Figure 7:**
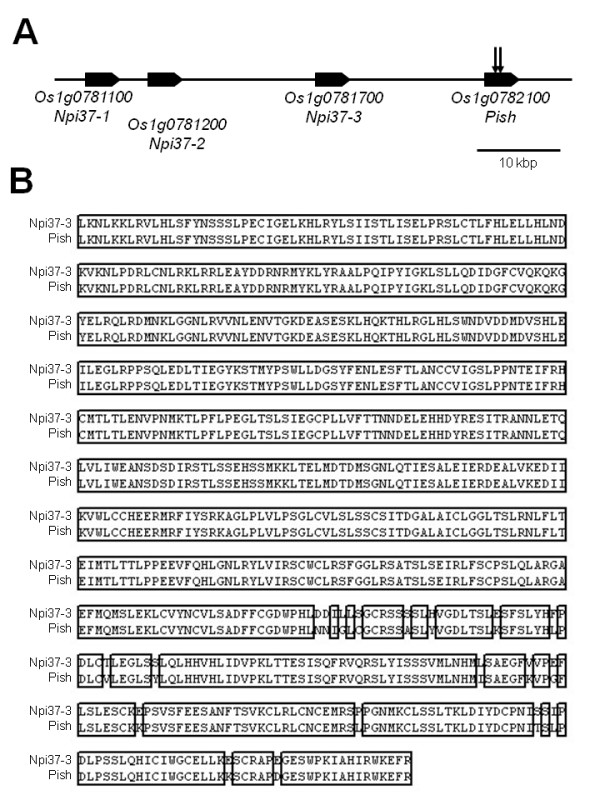
**Physical map of the region containing the *Pish *locus on chromosome 1**. (A) The *NLR *gene cluster in the *Pish *region. Arrows indicate the positions of the *Tos17 *insertions in the NC7869 and NC8589 lines. (B) Alignment of the LRR domains of Npi37-3 and Pish. Identical amino acid residues are enclosed by open boxes.

*Pish *(Os01g0782100) is present on the long-arm of chromosome 1. The RAP database revealed that three other NBS-LRR genes (Os01g0781100, Os01g0781200 and Os01g0781700) are arranged as tandem repeats near *Pish *within a 55-kb interval (Figure 7A). An allele of Os01g0781700 in the rice cv. St. No.1 encodes the R protein Pi37 [10]. Therefore, we named the genes *Npi37-1, Npi37-2 *and *Npi37-3*. Their deduced amino acid sequences show 67, 89, and 98% similarity, respectively, to Pish (Table 3). Npi37-3 shows the highest similarity to Pish, with differences at only 19 residues in the LRR domain (Figure 7B).

**Table 3:** Amino acid identity among the Npi37-1, Npi37-2, Npi37-3 and Pish proteins

	Npi37-1	Npi37-2	Npi37-3	Pish
Npi37-1		67	67	67
Npi37-2			91	89
Npi37-3				98

The amino acid sequences of the four NBS-LRR proteins were compared with each other using ClustalW http://www2.ebi.ac.uk/clustalw/.

### The *Pish *gene cluster is a hot spot of *Tos17 *insertions

**Figure 8:**
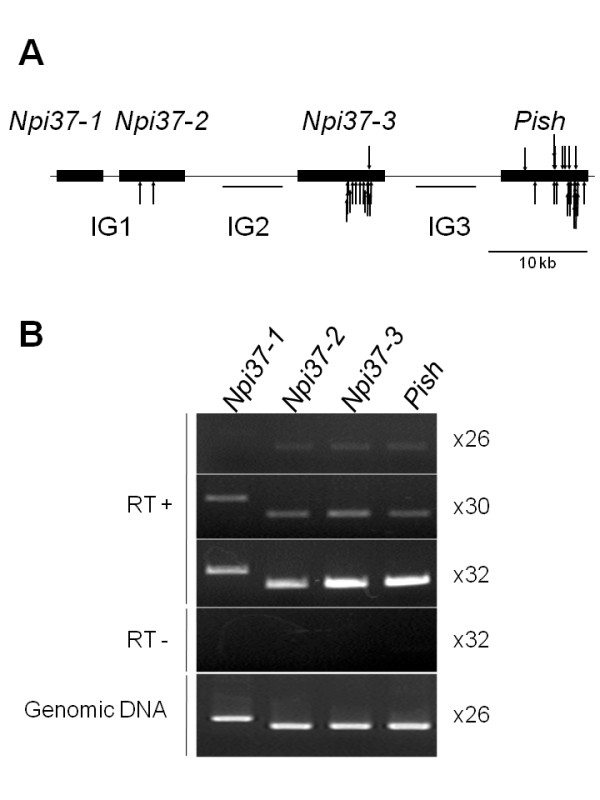
***Tos17 *insertions and expression of genes near the Pish locus**. (A) *Tos17 *insertion sites in the 55-kb genomic region containing the *Pish *locus are indicated by arrows. Downward arrows indicate insertions with *Tos17 *oriented in the same direction as *Pish *and the *Npi37-1 *to *Npi37-3 *genes. Upward arrows indicate insertions in the opposite orientation. The black boxes indicate genes. (B) RT-PCR analysis of the four *NLR *genes. One microgram of total RNA isolated from NB callus was used. A series of 26, 30, and 32 cycles of amplification was used for the PCR. The experiment was repeated three times, and typical results are shown. Genomic DNA isolated from NB callus was used as a control for PCR efficiency by each primer pair.

In this screening, we obtained 46 independent mutant alleles of *Pish *that were caused by *Tos17 *insertions. This suggested the possibility that the *Pish *locus is a hot spot of *Tos17 *insertions. It has been reported that *Tos17 *insertions are not distributed randomly in the rice genome, and several hot spots have been identified in NB, including genes encoding protein kinases and disease/defense-related genes [25]. An *in silico *analysis of the FST database revealed that 1,295 sequences flanking *Tos17 *insertion sites were present within the 55-kb genomic region including the four NBS-LRR genes (Table 4). These flanking sequences were particularly concentrated (1,294/1,295) within a region of about 47-kb that includes *Npi37-2*, *Npi37-3*, and *Pish*, but not the *Npi37-1 *locus. Based on the original callus lines and the sites of insertion, we determined that 193 of these insertions were independent. We also examined the 50-kb regions on either side of the 55-kb region. Even though each of these regions contains six predicted genes, the *Tos17 *insertions were highly concentrated in three of the four NBS-LRR genes (Table 4), indicating that these genes constitute a hot spot for *Tos17 *insertion. The four genes *Npi37-1*, *Npi37-2*, *Npi37-3*, and *Pish *contain highly similar sequences, and the sequences derived from FST are very short. Therefore, it was difficult to identify the exact site of each *Tos17 *insertion based on the FST sequences. To explore this further we randomly selected 50 independent insertions from the database and identified the insertion sites for 44 of them by PCR using specific primers. Unexpectedly, most of them were concentrated in either *Npi37-3 *or *Pish*. More than half of the insertions (27 of 44; 61%) were detected in the *Pish *gene, and 15 of 44 (34%) were in *Npi37-3 *(Figure 8A, Additional file 1). On the other hand, only two insertions were found in *Npi37-2 *and none were found in the *Npi37-1 *locus. This is surprising given that the *Npi37-2 *DNA sequence is 93% and 92% identical to those of *Npi37-3 *and *Pish*, respectively. *Tos17 *is activated by tissue culture, and we therefore speculated that the frequency of the insertion in a particular gene may depend on its expression level in cultured cells. To test this possibility, we analyzed the expression levels of each of the NBS-LRR genes in cultured cells. RT-PCR analysis demonstrated that the transcript levels of these four genes were not significantly different from one another (Figure 8B). These results suggest that neither gene structure nor relative expression levels are sufficient to explain the location of this hot spot.

**Table 4:** The numbers and positions of *Tos17 *insertions in a region of 155 kb including the *Pish *locus

Region	Independent^a^/FST database^b^	Length (bp)	Gene number
upstream region	0/0	50,000	6
*Npi37-1*	1/1	4,740	1
IG1^c^	0/0	1,711	0
*Npi37-2*	4-96/294*	6,778	1
IG2^c^	0/0	11,317	0
*Npi37-3*	0-168/500*	8,958	1
IG3^c^	0/0	11,721	0
*Pish*	20-180/500*	8,695	1
downstream region	39/76	50,000	6
Total	232 (193^d^)/1,371 (1,295^d^)	153,920	16

a) Number of non-redundant flanking sequences.

b) Number of flanking sequences described in the FST database including redundant sequences.

c) Intergenic regions between *Npi37-1 *and *Npi37-2 *(IG1), between *Npi37-2 *and *Npi37-3 *(IG2) and between *Npi37-3 *and *Pish *(IG3).

d) Number of flanking sequences in the 55 kb genomic region including *Npi37-1*, *Npi37-2*, *Npi37-3 *and *Pish*.

* Data including sequences whose exact positions were not determined.

The FST database also demonstrated that no *Tos17 *insertions occurred in the intergenic regions within this 55-kb genomic region (Table 4). The intergenic regions between *Npi37-2 *and *Npi37-3 *(IG2) and between *Npi37-3 *and *Pish *(IG3) are each more than 10 kb in length and show high levels of sequence similarity because of their duplication. An investigation of the intergenic regions using the RepeatMasker [35] program demonstrated that many ancient retrotransposon and other TE sequences are present in IG2 and IG3 (Additional file 2). In addition, a search of the rice MPSS database [36] indicated that the target sites of many small RNAs (sRNA) are present in IG2 and IG3 (data not shown). It is possible that these TE sequences constitute micro-heterochromatic regions due to the accumulation of these sRNA target sites. Because *Tos17 *insertions occur more frequently in gene-dense regions than in heterochromatin at the chromosomal level, this may explain why we found no *Tos17 *insertion in these intergenic regions.

## Discussion

### Large-scale screening using the retrotransposon *Tos17 *in rice

We screened 41,119 mutant lines induced by the rice retrotransposon *Tos17 *with the goal of obtaining signal components required for disease resistance in rice. This mutant population is estimated to carry over 400,000 insertions, based on our previous reports that the average number of insertions per line is about 10 [25,26]. We estimated that 400,000 insertions are required to provide a 99% chance of finding a mutant of any one gene [25,26]. Furthermore, since the lines were derived from cultured cells, they were expected to contain many deletions and substitutions (Table 1, [37]). Thus the number of mutant lines we employed here should have been enough for the identification of signaling mutants by genetic screening.

Among the mutations isolated by this screen, loss-of-function alleles of *Pish *were the most abundant. These mutants were easy to distinguish from wild-type plants because they exhibited complete susceptibility to a *M. oryzae *isolate containing *avrPish*. On the other hand, since the strength of resistance mediated by *Pish *is moderate, it was difficult to identify other mutations that caused reductions in resistance rather than a complete collapse in resistance. Another reason for the abundance of *pish *mutations in our screen is that the *Pish *locus is one of the hot spots for *Tos17 *insertions (Figure 8A). Therefore, it might have been inevitable that we would obtain a lot of *pish *mutants. The finding that most of the mutations isolated were loss-of-function alleles of an *R *gene is consistent with previous reports of screens for disease resistance mutations in Arabidopsis [38-40], tomato [41], and barley [42,43]. In Arabidopsis, Tornero and colleagues recovered 110 mutants that exhibited no HR after *avrRpm1 *induction, and 95 of them were loss-of-function mutations in the *R *gene *Rpm1*, which confers resistance to the bacterial pathogen *Pseudomonas syringae *pv. *tomato *expressing the *avrRpm1 *gene [40]. Jørgensen and colleagues performed a mutant screening of barley and isolated over 20 loss-of-function alleles of the *Mla12 *gene, which confers resistance to powdery mildew, and only three additional mutants in two other loci (*RAR1 *and *RAR2*) [43,44]. Thus, our ratio of 72 *pish *mutant lines to 14 other mutations was not surprising. The other 14 mutant lines have not yet been characterized, however, we expect that the analysis and isolation of the causative genes may provide novel insights into disease resistance signaling in rice.

### An NBS-LRR gene cluster in the *Pish *region

Genome sequencing revealed that there are up to 600 NBS-LRR genes in the rice genome and they account for about 1% of all of predicted ORFs [7]. The majority of *R *genes in the NBS-LRR class are physically clustered with closely related genes [45,46]. We found three additional NBS-LRR genes near *Pish*, arranged as tandem repeats (Figure 7A). Although one of these (*Npi37-3*) exhibits high similarity to *Pish*, with differences at only 19 residues in the LRR domain, it does not confer resistance to *M. oryzae *containing *avrPish *(Additional file 3). It is likely that some of these 19 residues are important for maintaining the target recognition site of Pish. This is consistent with reports that the LRR domain is required for the specific recognition of avirulence factors directly or indirectly, and that mutations in this domain often change gene-for-gene specificities [4,14]. On the other hand, the NBS domain may also be involved in the specific recognition of the target factors. *Npi37-3 *is an allele of another *R *gene, *Pi37 *[10]. The only difference between them is in two amino acid residues (V239A and I247M) in the NBS domain (Additional file 4). As discussed previously [10], these substitutions in the NBS domain are presumably involved in *avrPi37 *recognition.

*Pi35*(t) was identified in a QTL analysis of a population derived from the Japonica rice cultivar Hokkai 188 and the Indica rice cultivar Danghang-Shali. The gene was mapped on the long arm of chromosome 1 between the SSR markers RM1216 and RM1003, and is closely linked with *Pish *[47]. Interestingly, the resistance conferred by *Pi35*(t) to *M. oryzae *is classified as partial resistance (quantitative) rather than true resistance (qualitative). Although the race specificity and defense strength of *Pi35*(t) is different from that of *Pish*, it is possible that *Pi35*(t) is allelic to either *Pish *or *Pi37*. If so, the region containing the *Pish *locus is one of the clusters of *R *genes in rice. This is consistent with the idea that the evolution of *R *genes is driven by selection of allelic variations created by mutations or recombination within these clusters. This phenomenon has also been observed in the case of other *R *gene clusters such as the one containing *Pi9, Pi2 *and *Piz-t *on chromosome 6 [13,14]. The identification and characterization of *Pi35*(t) will allow us to understand the difference between partial and true resistance at the molecular level, and will also shed light on the evolution of these *R *genes during rice domestication.

### Evolution of the *Pish *gene cluster

Classical genetics and genome analysis have demonstrated that *R *genes tend to be clustered. The 55-kb region containing the *Pish *locus contains three other NBS-LRR genes, all oriented in the same direction (Figure 7A). Among the proteins encoded by these genes, Npi37-1 exhibits little similarity to the other three, while Npi37-3 shows high similarity to Npi37-2 (91%) and Pish (98%) (Table 3). Intriguingly, the N-terminal half of Npi37-3 is identical to that of Pish and the other half is identical to that of Npi37-2 (data not shown). These data suggest that at first the ancestral NBS-LRR gene was duplicated to produce *Npi37-1 *and *Npi37-2-pre*. This would be followed by a second duplication event in which *Npi37-2-pre *was duplicated to produce *Npi37-2 *and *Pish*. More recently, a crossover and/or duplication presumably occurred between *Npi37-2 *and *Pish*, resulting in *Npi37-2, Npi37-3*, and *Pish*. *Npi37-1 *and *Pish *are identical between the cultivars NB and St. No.1 [10], suggesting that the two paralogs, *Npi37-2 *and *Npi37-3*, probably mutated independently in NB and/or St. No.1 after the duplication events described above.

How did these frequent gene duplications in the *Pish *region occur? Although *R *gene loci in general tend to be duplicated, the mechanism for this duplication remains obscure. Gene duplication is sometimes caused by the misrepair of chromosomal double-strand breaks (DSBs), which arise spontaneously during the life of a cell. One possible inducer of DSBs is the endonuclease activity encoded by TEs. In *Drosphila melanogaster*, it has been reported that DSBs are important triggers of segmental duplication (SD), and the distribution of SDs correlates positively with that of TEs [48]. Here we showed that the *Pish *locus is one of the hot spots for *Tos17 *insertions (Figure 8). Therefore, the *Pish *locus was presumably attacked frequently by the *Tos17 *endonuclease, resulted in DSBs, which might have caused gene duplication. In addition to this effect of endonuclease activity, the insertion of TEs is thought to be important for the molecular evolution of *R *genes. Actually, many types of TEs have been identified in *R *gene clusters [22,23]. Recently, Hayashi and Yoshida [22,23] reported that the insertion of a retrotransposon *Renovator *in the promoter region of the blast *R *gene *Pit *promoted its expression and reactivation, demonstrating that the insertion of TEs has contributed to *R *gene evolution.

Our results indicating that the *Pish *locus is a hot spot for *Tos17 *insertions is consistent with a previous report that disease resistance genes are among the preferred targets for *Tos17 *insertion [25]. However, the disease resistance genes annotated in the report were predicted from their DNA sequence similarity with sequences encoding NBS and/or LRR domains, therefore there was no direct evidence indicating they truly function as *R *genes or components of defense signaling. Here, we demonstrated that the functional *R *gene *Pish *is actually a hot spot of *Tos17 *insertion in the NB genome, and is the preferred target site among four highly conserved and closely linked NBS-LRR genes, even though the genes are highly similar at both the nucleotide sequence and expression levels. These results suggest that *Tos17 *inserts most frequently in functional genes within hot spot regions. A search of the FST database indicates that not all NBS-LRR gene loci are hot spots for *Tos17 *insertion. It is possible that the NBS-LRR gene loci that are hot spots for TE insertions are functional *R *genes that have not yet been identified. Thus, it may be possible to predict novel functional *R *genes in the FST database by looking for regions that are hot spots for TE insertions. This possibility should be assessed in the future.

The molecular mechanisms that determine TE integration site specificity in plants are still unknown. Studies of the Ty retrotransposons of yeast have revealed that interactions with bound chromosomal proteins can tether the Ty integration machinery to chromosomes and thereby direct integration to nearby sites [49]. The human immunodeficiency virus (HIV) integrates preferentially into actively transcribed genes at sites with transcription-associated histone modifications. Therefore, it is possible that the insertion of *Tos17 *is regulated by chromatin structure or through interaction with chromatin binding proteins, rather than being controlled directly by the structures or expression levels of the targeted sequences.

## Conclusions

The genetic screening described here was a high-throughput system that allowed us to identify several mutants involved in *R *gene-mediated signal transduction and also to isolate the *R *gene *Pish*. We are confident that these mutants will be useful tools for the genetic analysis of defense signaling mechanism downstream of an *R *gene. Furthermore, our data provided experimental evidence that an *R *gene cluster is a highly preferred target for TE insertions. Our observations raise the hypothesis that recurrent gene duplication during the evolution of the *Pish *locus depended on this unique feature of being a preferred target site of *Tos17 *insertion. Further studies of the *Pish *locus will provide new insights into the functional and structural diversification of *R *genes, which is crucial for the survival of plants in their fight against rapidly evolving pathogens.

## Methods

### Plant and pathogen materials

The rice (*Oryza sativa*) cultivars Nipponbare (NB) and Kinmaze (KM) were used in this study. The mutant lines described in this article will be made available at the Rice Genome Resource Center of the National Institute of Agrobiological Sciences, Japan [50]. The *M. oryzae *isolate Kyu77-07A was used as an incompatible race against NB for our screening [19]. Other isolates, 84-81A (race 102.0), H07-1-1 (race 003.0), Kyu89-246 (race 003.0), Ina86-137 (race 007.0), TH68-126 (race 033.1), 24-22-1-1 (race 037.1), and Kyu9439013 (race 047.0), of which the country of origin is Japan, were used in complementation tests. *M. oryzae *was grown on oatmeal agar medium (30 g/l oatmeal, 5 g/l sucrose, and 16 g/l agar) at 22°C. Seedlings were inoculated at the 4-6 leaf stage by spraying to runoff with an aqueous spore suspension containing 1.5 × 10^5 ^spores per ml. Inoculated seedlings were kept in a dark chamber with a moisture-saturated atmosphere at 24°C for 20 h, and then maintained at 27°C and 70-80% relative humidity in a greenhouse. Disease development was monitored one week after inoculation.

### Co-segregation analysis and isolation of *Tos17 *flanking sequences

Linkage between the susceptible phenotype and transposed *Tos17 *fragments was analyzed by DNA gel blot hybridization or PCR. One microgram of rice genomic DNA was digested with *Xba*I, and the fragments were separated by electrophoresis in a 0.8% (w/v) agarose gel, then transferred to Amersham Hybond-N+ membranes (GE Healthcare). A 376 bp DNA probe specific to *Tos17 *was generated by digestion of a *Tos17 *genomic sequence with *EcoR*I and *BamH*I. A DNA probe specific to *Pish*(t) was generated by PCR using the primers AOL45 and AOL48 (Additional file 5). Sequences flanking *Tos17 *insertions were amplified by thermal asymmetric interlaced PCR (TAIL-PCR) as described previously [51]. Genotyping was performed by PCR using the *Pish*-specific primers AOL52 and AOL54 in combination with the *Tos17*-specific primer Tos17-448R (Additional file 5).

### Rice transformation

For complementation analyses, cDNA fragments corresponding to *Npi37-3 *and *Pish *were amplified by PCR using the primer sets AOL51-AOL53 and AOL51-AOL52 (Additional file 5). The coding sequences were cloned into the Ti-based vector pPZP2Ha3(+) downstream of the cauliflower mosaic virus 35 S promoter, and *Agrobacterium tumefaciens*-mediated transformation of rice callus was performed according to a published protocol [52,53]. Control calli were transformed with the empty vector. Plants regenerated from hygromycin-resistant calli were grown in an isolated greenhouse. More than five independent transgenic lines were produced for each construct. Expression of the transgenes and *Actin *(as a control) was confirmed by RT-PCR using the specific primer sets: AOL64-AOL53 for *Npi37-3*, AOL64-AOL52 for *Pish*, and OsAct1U- OsAct1L for *Actin *(Genbank accession number:AK100267).

### RNA Analysis

Poly(A)+ mRNA was purified using an Oligotex™-dT30 <Super> mRNA Purification kit (Takara) according to the manufacturers' instructions. As the probe, the DNA fragment corresponding to *Pish *was amplified by PCR from genomic DNA using the specific primer pairs AOL64-AOL71 (Additional file 5). The rice gene for *actin *was used as the loading control. Quantitative real-time RT-PCR was conducted using the iQ SYBR Green Supermix (BioRad, Hercules, CA, USA) and an iCycler (BioRad) according to the manufacturers' instructions. At least three independent biological samples were used with specific primers for each gene. The primers pairs were AOL351-AOL353 for *Pish *and OsAct1U- OsAct1L for an *actin *(Additional file 5). The data were normalized using *actin *gene expression levels. Gene expression in cultured cells was analyzed using specific primers for *Npi37-1 *(AOL212-AOL213), *Npi37-2 *(AOL234-AOL369), *Npi37-3 *(AOL62-AOL234), and *Pish *(AOL62-AOL233).

### Identification of *Tos17 *insertion sites

Genomic DNA was extracted from mutant lines selected from the flanking sequence tag (FST) database. *Tos17 *insertion sites were identified by PCR using specific primers for *Tos17 *(TAIL3 or T17-242R) in combination with the primers AOL71, AOL72, AOL356, or AOL357, which are specific for *Pish, Npi37-1, Npi37-2*, and/or *Npi37-3 *(Additional file 5).

## Authors' contributions

AT carried out all the genetic analyses, pathological tests, and molecular biology work, and participated in writing the first manuscript draft, and its revision. NH participated in the experimental design and provided expertise and all isolates of *M. oryzae*. AM provided all of the mutant seeds. HH conceived of the study, participated in the experimental design, participated in the coordination of the work, helped to draft the manuscript and contributed to its revision. All authors have read and approved the final manuscript.

## Supplementary Material

Additional file 1 **Insertion sites of *Tos17 *in the *Pish *locus**. The table lists the positions of the *Tos17 *insertion sites and their directions in the mutant lines. Click here for file

Additional file 2 **Investigation of the intergenic regions of the *Pish *locus**. The table lists ancient retrotransposon and transposon sequences presented within the 55-kb genomic region including the *Pish *locus. SW score: Smith-Waterman score of the match, usually complexity adjusted. perc div.: % substitutions in matching region compared with the consensus. perc del.: % of bases opposite a gap in the query sequence (deleted bp). perc ins.: % of bases opposite a gap in the repeat consensus (inserted bp). position in query: starting or ending position of match in query sequence. direction: (C) = match is with the Complement of the consensus sequence in the database. matching repeat: name of the matching interspersed repeat. repeat class/family: the class of the repeat Click here for file

Additional file 3 **Complementation tests of *Npi37-3 *transgenic plants**. A cDNA of *Npi37-3 *under the control of the 35 S promoter was introduced into KM. The empty vector was used as a control. The photographs show leaf blades 7 days after the plants were inoculated as described for the experiment shown in Figure 5. The expression of the transgene was confirmed by RT-PCR analysis. An *Actin *gene was used as a control for RNA template amounts. Click here for file

Additional file 4 **Alignment of the predicted amino acid sequences of Pish, Npi37-3, and Pi37**. Identical amino acid residues are enclosed by open boxes. Stars indicate two amino acid residues that differ between Npi37-3 and Pi37. Click here for file

Additional file 5 **Primer pairs utilized in this work**. The table lists the primers sequence with their direction and target genes. Click here for file
