# Supplementary material for: Unique features of the rice blast resistance Pish locus revealed by large scale retrotransposon-tagging
Source: BMC Plant Biol. 2010 Aug 13;10:175. doi: 10.1186/1471-2229-10-175 (PMC3017791; doi:10.1186/1471-2229-10-175)
Supplement: Additional file 1 — Insertion sites of Tos17 in the Pish locus. The table lists the positions of the Tos17 insertion sites and their directions in the mutant lines. [file 1471-2229-10-175-S1.PDF]

Locus	Line Name	Position[a]	Direction[b]
Npi37-2	NF2014	34855844	↑
	NE3035	34856261	↑
Npi37-3	NF2038	34876293	↑
	NF6826	34876839	↑
	NF2780	34877136	↑
	NE1044	34877345	↑
	NF6012	34877775	↑
	NE4526	34877931	↑
	NF9843	34878143	↑
	NF7803	34878261	↑
	NE8544	34878280	↓
	NE6027	34878996	↑
	NF6805	34879264	↑
	NE8539	34879414	↑
	ND9015	34879543	↑
	NF2753	34879819	↑
	ND9042	34879845	↑
Pish	ND7033	34895803	↓
	NE1018	34897973	↑
	NE3050	34898316	↑
	NE7012	34898342	↓
	NF1034	34898430	↓
	NF7813	34898631	↑
	NE5044	34898859	↓
	NE7001	34898981	↓
	NE0006	34899268	↑
	NE1544	34899323	↓
	NF6036	34899425	↑
	NE0014	34899470	↑
	NE1520	34899483	↑
	ND7070	34899559	↓
	NE4034	34899563	↑
	NE8006	34899697	↑
	ND8008	34899751	↑
	NF9014	34899892	↑
	NE5001	34900167	↓
	NF9012	34900218	↑
	NE4502	34900221	↑
	NF9013	34900386	↑
	NF1031	34900390	↑
	NE4038	34900447	↑
	ND9006	34900776	↑
	ND8036	34900920	↑
	NF4907	34901295	↑

a) Position of the *Tos17* insertion on chromosome 1
b) The upward arrows indicate insertions of *Tos17* in the opposite direction of *Npi37-2* , *Npi37-3,* or *Pish*.
