# Supplementary material for: Unique features of the rice blast resistance Pish locus revealed by large scale retrotransposon-tagging
Source: BMC Plant Biol. 2010 Aug 13;10:175. doi: 10.1186/1471-2229-10-175 (PMC3017791; doi:10.1186/1471-2229-10-175)
Supplement: Additional file 2 — Investigation of the intergenic regions of the Pish locus. The table lists ancient retrotransposon and transposon sequences presented within the 55-kb genomic region including the Pish locus. SW score: Smith-Waterman score of the match, usually complexity adjusted. perc div.: % substitutions in matching region compared with the consensus. perc del.: % of bases opposite a gap in the query sequence (deleted bp). perc ins.: % of bases opposite a gap in the repeat consensus (inserted bp). position in query: starting or ending position of match in query sequence. direction: (C) = match is with the Complement of the consensus sequence in the database. matching repeat: name of the matching interspersed repeat. repeat class/family: the class of the repeat [file 1471-2229-10-175-S2.PDF]

	SW score	perc div.	perc del.	perc ins.	position in query begin	end	direction	matching repeat	repeat class/family
Npi37-1									
IG1	2285	12.6	0.3	0.3	34852240	34852589	C	SINE03_OS	SINE
Npi37-2									
IG2	2202	7.9	1.6	1.3	34859925	34860231	C	COPI1_LTR	LTR/Copia
	1225	15.8	1.1	0.4	34861358	34861617	C	STOWAWAY41_OS	DNA/TcMar-Stowaway
	1699	21.4	3.4	1.2	34863939	34864354	+	MERMITEA	DNA
	5388	11.8	4.3	3.1	34864904	34865805	C	SZ-44_LTR	LTR/Gypsy
	3085	20.9	7	7.3	34865806	34867374	+	SZ-40_LTR	LTR/Gypsy
	15727	5.2	0.5	1.6	34867347	34869349	+	SZ-40_LTR	LTR/Gypsy
	2556	12.6	1.1	4.5	34869350	34869778	C	SZ-21_LTR	LTR/Gypsy
	1470	19.9	3.2	3.2	34869977	34870319	C	MERMITED	DNA
	1665	15.1	7	1.1	34870483	34870838	+	MERMITE18B	DNA
	1480	18.5	7	1.3	34871620	34871976	C	MERMITE18B	DNA
Npi37-3									
IG3	2202	7.9	1.6	1.3	34880553	34880859	C	COPI1_LTR	LTR/Copia
	1201	16.2	1.1	0.4	34881986	34882245	C	STOWAWAY41_OS	DNA/TcMar-Stowaway
	1699	21.4	3.4	1.2	34884566	34884981	+	MERMITEA	DNA
	5388	11.8	4.3	3.1	34885531	34886432	C	SZ-44_LTR	LTR/Gypsy
	3079	21	6.8	7.2	34886433	34888002	+	SZ-40_LTR	LTR/Gypsy
	15727	5.2	0.5	1.6	34887975	34889977	+	SZ-40_LTR	LTR/Gypsy
	2556	12.6	1.1	4.5	34889978	34890406	C	SZ-21_LTR	LTR/Gypsy
	1470	19.9	3.2	3.2	34890605	34890947	C	MERMITED	DNA
	1665	15.1	7	1.1	34891111	34891466	+	MERMITE18B	DNA
	1480	18.5	7	1.3	34892248	34892604	C	MERMITE18B	DNA
Pish									
