# Supplementary figures and images for: Unique features of the rice blast resistance Pish locus revealed by large scale retrotransposon-tagging
Source: BMC Plant Biol. 2010 Aug 13;10:175. doi: 10.1186/1471-2229-10-175 (PMC3017791; doi:10.1186/1471-2229-10-175)

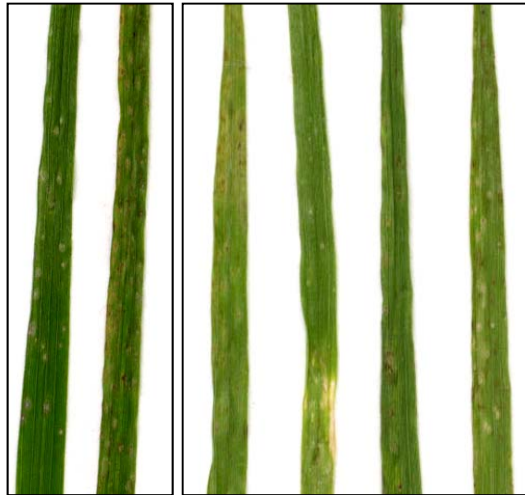

|     |                |                |                 |                |
|-----|----------------|----------------|-----------------|----------------|
|     | A <sub>2</sub> | A <sub>3</sub> | A <sub>14</sub> | A <sub>4</sub> |
| Vec | Npi37-3        |                |                 |                |

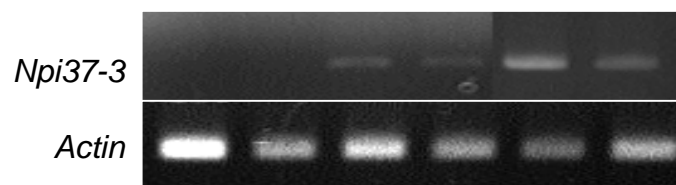

Supplement: Additional file 3 — Complementation tests of Npi37-3 transgenic plants. A cDNA of Npi37-3 under the control of the 35 S promoter was introduced into KM. The empty vector was used as a control. The photographs show leaf blades 7 days after the plants were inoculated as described for the experiment shown in Figure 5. The expression of the transgene was confirmed by RT-PCR analysis. An Actin gene was used as a control for RNA template amounts. [file 1471-2229-10-175-S3.PDF]
