# Supplementary material for: Unique features of the rice blast resistance Pish locus revealed by large scale retrotransposon-tagging
Source: BMC Plant Biol. 2010 Aug 13;10:175. doi: 10.1186/1471-2229-10-175 (PMC3017791; doi:10.1186/1471-2229-10-175)
Supplement: Additional file 4 — Alignment of the predicted amino acid sequences of Pish, Npi37-3, and Pi37. Identical amino acid residues are enclosed by open boxes. Stars indicate two amino acid residues that differ between Npi37-3 and Pi37. [file 1471-2229-10-175-S4.PDF]

```
*        *
Pish     MAEVVLAGLR LAATPICVKL LCNASTCLGV DMTRELHELE TIIIPQFELV IEAAEKGNHR AKLDRWLREL KQAFYNAEDL LDEHEYNILK CKAKHKDSLV  100
Npi37-3  MAEVVLAGLR LAATPICVKL LCNASTCLGV DMTRELHELE TIIIPQFELV IEAAEKGNHR AKLDRWLREL KQAFYNAEDL LDEHEYNILK CKAKHKDSLV  100
Pi37     MAEVVLAGLR LAATPICVKL LCNASTCLGV DMTRELHELE TIIIPQFELV IEAAEKGNHR AKLDRWLREL KQAFYNAEDL LDEHEYNILK CKAKHKDSLV  100

Pish     KDSTQVHDSS ISNILKQPMR AVSSRMSNLR PENRKILCQL NELKTMLEKA KEFRELIHLP AGNSLEGPSV PTIVVPVVTS LLPPRVFGRN MDRDRIIHLL  200
Npi37-3  KDSTQVHDSS ISNILKQPMR AVSSRMSNLR PENRKILCQL NELKTMLEKA KEFRELIHLP AGNSLEGPSV PTIVVPVVTS LLPPRVFGRN MDRDRIIHLL  200
Pi37     KDSTQVHDSS ISNILKQPMR AVSSRMSNLR PENRKILCQL NELKTMLEKA KEFRELIHLP AGNSLEGPSV PTIVVPVVTS LLPPRVFGRN MDRDRIIHLL  200

                                                     *          *
Pish     TKPMATVSSS VGYSGLAIVA HGGAGKSTLA QCVYNDKRVQ EHFDVRIWVC ISRKLDVHRH TREIIESATN GECPRVDNLD TLQCRLKDIM QKSEKFLLVL  300
Npi37-3  TKPMATVSSS VGYSGLAIVA HGGAGKSTLA QCVYNDKRVQ EHFDVRIWVC ISRKLDVHRH TREIIESATN GECPRVDNLD TLQCRLKDIM QKSEKFLLVL  300
Pi37     TKPMATVSSS VGYSGLAIVA HGGAGKSTLA QCVYNDKRAQ EHFDVRMWVC ISRKLDVHRH TREIIESATN GECPRVDNLD TLQCRLKDIM QKSEKFLLVL  300

Pish     DDVWFDESVN EREWDQLLDP LVSQQEGSRV LVTSRRDVLP AALHCKDVVH LENMEDAEFL ALFKYHAFSG TEIRNPQLHA RLEEVAEKIA KRLGQSPLAA  400
Npi37-3  DDVWFDESVN EREWDQLLDP LVSQQEGSRV LVTSRRDVLP AALHCKDVVH LENMEDAEFL ALFKYHAFSG TEIRNPQLHA RLEEVAEKIA KRLGQSPLAA  400
Pi37     DDVWFDESVN EREWDQLLDP LVSQQEGSRV LVTSRRDVLP AALHCKDVVH LENMEDAEFL ALFKYHAFSG TEIRNPQLHA RLEEVAEKIA KRLGQSPLAA  400

Pish     RTVGSQLSRN KDIAIWKSAL NIENLSEPMK ALLWSYNKLD SRLQRCFLYC SLFPKGHKYK IDEMVDLWVA EGLVDSRNQG DKRIEDIGRD YFNEMVSGSF  500
Npi37-3  RTVGSQLSRN KDIAIWKSAL NIENLSEPMK ALLWSYNKLD SRLQRCFLYC SLFPKGHKYK IDEMVDLWVA EGLVDSRNQG DKRIEDIGRD YFNEMVSGSF  500
Pi37     RTVGSQLSRN KDIAIWKSAL NIENLSEPMK ALLWSYNKLD SRLQRCFLYC SLFPKGHKYK IDEMVDLWVA EGLVDSRNQG DKRIEDIGRD YFNEMVSGSF  500

Pish     FQPVSERYMG TWYIMHDLLH DLAESLTKED CFRLEDDGVK EIPATVRHLS ICVDSMKFHK QKICKLRYLR TVICIDPLMD DGDDIFNQLL KNLKKLRVLH  600
Npi37-3  FQPVSERYMG TWYIMHDLLH DLAESLTKED CFRLEDDGVK EIPATVRHLS ICVDSMKFHK QKICKLRYLR TVICIDPLMD DGDDIFNQLL KNLKKLRVLH  600
Pi37     FQPVSERYMG TWYIMHDLLH DLAESLTKED CFRLEDDGVK EIPATVRHLS ICVDSMKFHK QKICKLRYLR TVICIDPLMD DGDDIFNQLL KNLKKLRVLH  600

Pish     LSFYNSSSLP ECIGELKHLR YLSIISTLIS ELPRSLCTLF HLELLHLNDK VKNLPDRLCN LRKLRRLEAY DDRNRMYKLY RAALPQIPYI GKLSLLQDID  700
Npi37-3  LSFYNSSSLP ECIGELKHLR YLSIISTLIS ELPRSLCTLF HLELLHLNDK VKNLPDRLCN LRKLRRLEAY DDRNRMYKLY RAALPQIPYI GKLSLLQDID  700
Pi37     LSFYNSSSLP ECIGELKHLR YLSIISTLIS ELPRSLCTLF HLELLHLNDK VKNLPDRLCN LRKLRRLEAY DDRNRMYKLY RAALPQIPYI GKLSLLQDID  700

Pish     GFCVQKQKGY ELRQLRDMNK LGGNLRVVNL ENVTGKDEAS ESKLHQKTHL RGLHLSWNDV DDMDVSHLEI LEGLRPPSQL EDLTIEGYKS TMYPSWLLDG  800
Npi37-3  GFCVQKQKGY ELRQLRDMNK LGGNLRVVNL ENVTGKDEAS ESKLHQKTHL RGLHLSWNDV DDMDVSHLEI LEGLRPPSQL EDLTIEGYKS TMYPSWLLDG  800
Pi37     GFCVQKQKGY ELRQLRDMNK LGGNLRVVNL ENVTGKDEAS ESKLHQKTHL RGLHLSWNDV DDMDVSHLEI LEGLRPPSQL EDLTIEGYKS TMYPSWLLDG  800

Pish     SYFENLESFT LANCCVIGSL PPNTEIFRHC MTLTLENVPN MKTLPFLPEG LTSLSIEGCP LLVFTTNNDE LEHHDYRESI TRANNLETQL VLIWEANSDS  900
Npi37-3  SYFENLESFT LANCCVIGSL PPNTEIFRHC MTLTLENVPN MKTLPFLPEG LTSLSIEGCP LLVFTTNNDE LEHHDYRESI TRANNLETQL VLIWEANSDS  900
Pi37     SYFENLESFT LANCCVIGSL PPNTEIFRHC MTLTLENVPN MKTLPFLPEG LTSLSIEGCP LLVFTTNNDE LEHHDYRESI TRANNLETQL VLIWEANSDS  900

Pish     DIRSTLSSEH SSMKKLTELM DTDMSGNLQT IESALEIERD EALVKEDIIK VWLCCHEERM RFIYSRKAGL PLVLPSGLCV LSLSSCSITD GALAICLGGL  1000
Npi37-3  DIRSTLSSEH SSMKKLTELM DTDMSGNLQT IESALEIERD EALVKEDIIK VWLCCHEERM RFIYSRKAGL PLVLPSGLCV LSLSSCSITD GALAICLGGL  1000
Pi37     DIRSTLSSEH SSMKKLTELM DTDMSGNLQT IESALEIERD EALVKEDIIK VWLCCHEERM RFIYSRKAGL PLVLPSGLCV LSLSSCSITD GALAICLGGL  1000

Pish     TSLRNLFLTE IMTLTTLPPE EVFQHLGNLR YLVIRSCWCL RSFGGLRSAT SLSEIRLFSC PSLQLARGAE FMQMSLEKLC VYNCVLSADF FCGDWPHLNN  1100
Npi37-3  TSLRNLFLTE IMTLTTLPPE EVFQHLGNLR YLVIRSCWCL RSFGGLRSAT SLSEIRLFSC PSLQLARGAE FMQMSLEKLC VYNCVLSADF FCGDWPHLDD  1100
Pi37     TSLRNLFLTE IMTLTTLPPE EVFQHLGNLR YLVIRSCWCL RSFGGLRSAT SLSEIRLFSC PSLQLARGAE FMQMSLEKLC VYNCVLSADF FCGDWPHLDD  1100

Pish     IGLQGCRSSA SLYVGDLTSL KSFSLYHLPD LCVLEGLSYL QLHHVHLIDV PKLTTESISQ FRVQRSLYIS SSVMLNHMIS AEGFKVPGFL SLESCKKPSV  1200
Npi37-3  ILLSGCRSSS SLHVGDLTSL ESFSLYHFPD LCTLEGLSSL QLHHVHLIDV PKLTTESISQ FRVQRSLYIS SSVMLNHMLS AEGFVVPEFL SLESCKEPSV  1200
Pi37     ILLSGCRSSS SLHVGDLTSL ESFSLYHFPD LCTLEGLSSL QLHHVHLIDV PKLTTESISQ FRVQRSLYIS SSVMLNHMLS AEGFVVPEFL SLESCKEPSV  1200

Pish     SFEESANFTS VKCLRLCNCE MRSLPGNMKC LSSLTKLDIY DCPNITSLPD LPSSLQHICI WGCELLKKSC RAPDGESWPK IAHIRWKEFR  1290
Npi37-3  SFEESANFTS VKCLRLCNCE MRSPPGNMKC LSSLTKLDIY DCPNISSIPD LPSSLQHICI WGCELLKESC RAPEGESWPK IAHIRWKEFR  1290
Pi37     SFEESANFTS VKCLRLCNCE MRSPPGNMKC LSSLTKLDIY DCPNISSIPD LPSSLQHICI WGCELLKESC RAPEGESWPK IAHIRWKEFR  1290
```
