# Supplementary material for: Unique features of the rice blast resistance Pish locus revealed by large scale retrotransposon-tagging
Source: BMC Plant Biol. 2010 Aug 13;10:175. doi: 10.1186/1471-2229-10-175 (PMC3017791; doi:10.1186/1471-2229-10-175)
Supplement: Additional file 5 — Primer pairs utilized in this work. The table lists the primers sequence with their direction and target genes. [file 1471-2229-10-175-S5.PDF]

Additional file 5

Primer pairs utilized in this work

Primers	Sequence	direction	target
AOL45	TCAGAAATCCACAGTTGCATGCAC	Forward	*Npi37-3* and *Pish*
AOL48	GGAGAGCTGCTCTATACAATTTATAC	Reverse	*Npi37-3* and *Pish*
AOL51	AGCTGCAGTAGTGCTGTTCCATG	Forward	*Npi37-3* and *Pish*
AOL52	CGTAAGATCATGAGCGAATG	Reverse	*Pish*
AOL53	TGCATGATCATGGGTTAACG	Reverse	*Npi37-3*
AOL54	GTGAAAGATTCCACCCAGGTTC	Forward	*Pish*
AOL62	TACTCTCTCTTTCCTCCTGTAG	Forward	*Npi37-3* and *Pish*
AOL64	TCCAAAGCTTACTACCGAGAGC	Forward	*Npi37-2, Npi37-3* and *Pish*
AOL71	TTGCGCTTCTCCGTAGTAGTG	Reverse	*Pish*
AOL72	TTCCGTTTCTCTCTAGTTGTG	Reverse	*Npi37-2* and *Npi37-3*
AOL212	ACAGCACCCATCAGGCCTAAC	Forward	*Npi37-1*
AOL213	GCGGGACGTTACTAGAATTTTG	Reverse	*Npi37-1*
AOL233	GCTTCTACACCCACATAACCC	Reverse	*Pish*
AOL234	GCTTCTGCACCCAGATAAGAG	Reverse	*Npi37-2* and *Npi37-3*
AOL351	ACTGCCCCAACATAACATCTC	Forward	*Pish*
AOL353	CCTGCAGCTCTTCTTCAAAAG	Reverse	*Pish*
AOL356	GACGGAGGGAGTATGTCTAAC	Forward	*Npi37-3* and *Pish*
AOL357	TTTAACAACGTCCCACTTGTC	Forward	*Npi37-2*
AOL369	AACTCAATCTTTTCTCCTGTAG	Forward	*Npi37-2*
Tos17-448R	CGTTGCTACATCTACCAGTTCG	Reverse	*Tos17*
TAIL3	GAGAGCATCATCGGTTACATCTTCTC	Forward	*Tos17*
T17-242R	CTTAACCTCCTCCAACGAATCG	Reverse	*Tos17*
OsAct1U	TCCATCTTGGCATCTCTCAG	Forward	*Actin*
OsAct1L	GTACCCGCATCAGGCATCTG	Reverse	*Actin*
